# Comparison of Clinicopathological Features and Treatments between Young (≤40 Years) and Older (>40 Years) Female Breast Cancer Patients in West China: A Retrospective, Epidemiological, Multicenter, Case Only Study

**DOI:** 10.1371/journal.pone.0152312

**Published:** 2016-03-31

**Authors:** Ke Wang, Yu Ren, Hongyuan Li, Ke Zheng, Jun Jiang, Tianning Zou, Binlin Ma, Hui Li, Qilun Liu, Jianghua Ou, Ling Wang, Wei Wei, Jianjun He, Guosheng Ren

**Affiliations:** 1 Department of Breast Surgery, the First Affiliated Hospital of Xi’an Jiaotong Universtiy, Xi’an, Shaanxi Province, China; 2 Department of Endocrine and Breast Surgery, the First Affiliated Hospital of Chongqing Medical University, Chongqing, China; 3 Department of Breast Disease Center, Third Military Medical University, Chongqing, China; 4 Breast Cancer Research Center, Yunnan Tumor Hospital, Kunming, Yunnan Province, China; 5 Department of Breast and Neck Surgery, Affiliated Tumor Hospital of Xinjiang Medical University, Urumqi, Xinjiang Province, China; 6 Department of Breast Surgery, Sichuan Province Tumor Hospital, Chengdu, Sichuan Province, China; 7 Department of Surgical Oncology, Affiliated Hospital of Ningxia Medical University, Yinchuan, Ningxia Province, China; 8 Department of Breast Surgery, Affiliated Tumor Hospital of Xinjiang Medical University, Urumqi, Xinjiang Province, China; 9 Department of Vascular and Endocrine Surgery, Xijing Hospital of Fourth Military Medical University, Xi’an, Shaanxi Province, China; 10 Department of Breast Surgery, Cancer Hospital of Guangxi Medical University, Nanning, Guangxi Province, China; INRS, CANADA

## Abstract

The incidence of young cases of breast cancer is higher in China compared to the western world. We aimed to explore differences in risk factors, clinicopathological features and treatment modes of young female breast cancer compared to older patients in West China. We collected clinical information from 12,209 female breast cancer patients in West China, including risk factors, clinicopathological features and treatment modes, from January 2010 to December 2012. Chi-square tests and the multivariate logistic regression analysis were applied for statistical analysis. There were 2,682 young (≤40 years) cases and 9,527 older cases at the time of breast cancer diagnosis. Young patients had a greater tumor diameter at diagnosis, and a higher probability of axillary lymph node and distant metastasis (P < 0.05). The progesterone receptor positive expression rate, estrogen receptor/progesterone receptor double positive expression rate, and human epidermal growth factor receptor 2 (HER2) negative expression rate was higher in young patients compared to older patients (P < 0.05). For young patients, the age at menarche was earlier, they had lower marriage rates, fewer pregnancies and births, and a lower breastfeeding rate (P < 0.05). A higher proportion of young patients underwent advanced operations, neoadjuvant and adjuvant chemotherapy, radiotherapy, and endocrine therapy compared to older patients (P < 0.05). We found significant differences in the clinicopathological features, risk factors and treatment modes between young (≤40 years) and older (>40 years) female breast cancer patients in West China. As some of these results differ from those found in the western female population, it is likely that the mechanism of tumorigenesis of young female breast cancer patients in West China may differ from that in western developed countries. Further investigation into the regional differences in breast cancer tumorigenesis is warranted.

## Introduction

Differences in the clinical and biological characteristics between young and older breast cancer patients have been observed. Breast cancer in young patients is often more malignant, at a later tumor stage with higher tumor grade, has a larger tumor size, and there are more cases that are also lymph node positive compared to older patients [1–3]. Therefore, different treatment strategies should be adopted to treat young and older breast cancer patients, respectively.

In recent years, the incidence of breast cancer has leaped to the number one female cancer in China. In 2010, the crude incidence rate of female breast cancer patients were 32.43 per 100,000 and the estimated number of death was about 55,500 in China.[4] Several studies have shown that young patients (≤40 years) accounted for about 20% of the total breast cancer cases in China [5, 6], which is significantly higher than that of western population (i.e., around 5%) [7, 8]. This suggests the possibility that certain differences in the pathogenesis of breast cancer may exist between young Chinese women and women in the western population.

Despite the large number of cases of young female breast cancer (≤40 years) in China, few multicenter studies have been performed correlating onset age with clinicopathological features and treatment mode. This study aimed to retrospectively investigate the morbidity of female breast cancer in nine provinces of West China, and compare the differences in clinicopathological features and treatment options between young and older female breast cancer patients. The results of this study will provide new reference data for developing more suitable clinical diagnosis and treatment strategies for female breast cancer in West China. In addition, as West China is typical of a developing economic region, our results may also provide reference data for new diagnosis and treatment strategies for breast cancer in other developing countries.

## Methods

### Study design

The Western China Clinical Cooperation Group (WCCCG) was established in 2008 and includes 23 breast cancer centers from nine provinces of West China (i.e., Chongqing, Sichuan, Yunnan, Guizhou, Shaanxi, Ganshu, Guangxi, Ningxia and Xinjiang).The Western Chinese breast cancer multi-center clinical epidemiological study is a hospital based, multi-center, 3-year retrospective study of randomly selected pathology confirmed primary female breast cancer cases via medical chart review.

### Selection of regions and hospitals

Almost all of western provinces are involved in this study except Tibet and Qinghai, because of small population and low diagnosis and treatment ability of breast cancer in these two areas. Then, 2~3 representative hospitals were selected from every province. Qualified participating hospitals should have the following conditions: (1) they are one of the best leading hospital of the tertiary level and are regional referral centers providing pathology diagnosis, surgery, radiotherapy, medical oncology, and routine follow-up care for patients with breast cancer; (2) they can represent for the regional patients resource; and (3) the breast cancer screening practices, when used, should be in accordance with Chinese national standards [6]. A total of 23 hospitals are involved in the study, with the First Affiliated Hospital of Chongqing Medical University as the lead centre for the overall coordination of the implementation of this research in West China. These centers are representative of cancer therapeutic centers in the region, and therefore, cases from these centers allow us to determine the characteristics and treatment status of female breast cancer in West China.

### Patients

This study includes pathology confirmed female primary breast cancer inpatients in every month, except January and February, from year 2010 to 2012, because Chinese traditional spring festival is always in these two months and there are much fewer inpatients during the time period. In order to avoid selection bias, 20 patients’ medical records were randomly collected from each hospital in each month according to enrolment scheme. Because huge difference in regional population could lead to a different number of new cases of breast cancer, some hospitals in small population regions could not undertake collection of more than 20 patients’ records every month. All appropriate cases were reviewed and patient’s information was collected based on the designed case report form. In each month, if inpatients admissions are less than 20, more cases from the neighboring months will be reviewed until the total number in that year reaches 200. To ensure that the study is geographically representative, it was designed to include patients enrolled at sites from almost all of western provinces in China.

Patient identity information was removed from all collected information and patient records were analyzed anonymously. The inclusion criteria for this study were: (1) female breast cancer patients confirmed by pathological diagnosis; and (2) had received breast cancer-related treatment. The exclusion criteria were: (1) male breast cancer patients; and (2) an initial diagnosis age of more than 100 years, or less than 16 years.

### Ethics statement

The study was approved by the Institutional Review Board of WCCCG. The institutional review board obtained written consent from participating hospitals to access patient medical records. As it was a 3-year retrospective design, some data subjects were deceased, and it was impossible to contact with the patients or their relatives so the institutional review board of WCCCG waived the need for written informed consent from the participants.

### Data collection

According to a predetermined recruitment plan, we collected more than 12,000 qualified female breast cancer patients from every medical center from 2010 to 2012 (About 10% of original data in S1 Dataset). Two data input clerks in each center were responsible for entering patient information into medical records. Every step in the information collection process was under quality control. The organizational unit (i.e., the First Affiliated Hospital of Chongqing Medical University) summarized all data from each center. EpiData (http://www.epidata.dk/) software was used to perform data validation and a consistency check. For a detailed method of the data collection process, see Li et al. [6].

The following data was systematically collected for all enrolled patients via medical chart review: (1) general information including date of diagnosis, visits to other health care professionals, inpatient admission date, diagnosis at admission, inpatient discharge date, discharge outcome; (2) demographic characteristics at the time of diagnosis/admission, including age, body weight and height, and so on; (3) data from the clinical breast examination; (4) data from diagnostic imaging, including mammography and ultrasound; (5) data on the use of currently available surgery approaches; (6) data on the use of radiotherapy for breast cancer; (7) data on the use of chemotherapy for breast cancer, including adjuvant chemotherapy and neoadjuvant chemotherapy; (8) data on the use of endocrine therapy for breast cancer; (9) data on risk factors; (10) data on the pathological characteristics, including pre-surgery cytology and pathology examinations, intraoperative pathology evaluation, post-surgery pathology, estrogen and progesterone receptor expression, human epidermal growth factor receptor 2 (HER2) expression, and so on.

### Pathological diagnostic criteria

Histological subtype was based on the 1981 and 2003 world health organization (WHO) histological classification criteria [9, 10]. Staging of breast cancer was performed according to the American Joint Committee on Cancer (AJCC) TNM staging system (from 1997 and 2002) [11, 12].

### Definition of "young"

There is still controversy on how best to define “young” breast cancer patients. Zhou and Recht [13] researched the definition of “young age” by searching MEDLINE and Cancer Lit databases. They found that women “35 to 40 years of age or younger” defined a group of patients in which age was an independent risk factor for higher rates of recurrence of invasive breast cancer. Accordingly, patients that were aged ≤40 years are considered “young patients”. Therefore, in this study, we divided all patients into either a “young” ≤40 years group and an “older” >40 years group.

### Statistical analysis

Frequencies were run on variables related to the clinical and pathological characteristics, risk factors, and various treatment modes, to determine their distribution overall and among the different age groups. The differences in distribution of variables between the different age groups were examined using the Mantel-Haenszel chi-square tests to obtain p-values for the test of no-association. The multivariate logistic regression analysis was used to identify the estrogen-related risk factors for “young” age breast cancer. The odds ratio (OR) with 95% confidence intervals were also calculated. SPSS statistical software version 17.0 (SPSS Inc. Chicago, IL, USA) was used to analyze the data. Statistical significance was assessed by two-tailed tests with α level of 0.05.

## Results

### General information of patients

A total of 12,209 patients were enrolled in this study; 2,682 cases (i.e., 21.97%) were ≤40 years old at the time of diagnosis. The median diagnosis age of all patients was 48 years.

### Comparison of breast cancer risk factors between the two age groups

We analyzed the distribution of estrogen-related breast cancer risk factors as these factors may affect both the pathogenesis of breast cancer and the expression level of hormone receptors (Table 1). We found that the age of menarche was significantly earlier (p < 0.001) in the young (≤40 years) patients compared to older (>40 years) patients. Young patients also showed a significantly lower (p < 0.001) marriage rate, numbers of pregnancies and births, and a lower breastfeeding rate than the older group of breast cancer patients.

**Table 1:** Comparison of breast cancer risk factors between young (≤40 years) and older (>40 years) female breast cancer patients.

Characteristic	≤40 years		>40 years		P-value
	N	percent	N	percent	
**Age at menarche (years)**					<0.001
≤10	7	0.26	12	0.13	
11–12	421	15.70	1052	11.04	
13–14	1514	56.45	4439	46.59	
15–16	586	21.85	2774	29.12	
17–18	128	4.77	1001	10.51	
≥19	19	0.71	224	2.35	
Missing data	7	0.26	25	0.26	
Total	2682		9527		12209
**Marital status**					<0.001
Married	2592	96.64	9313	97.75	
Never married/single	63	2.35	26	0.27	
Divorced/widow	23	0.86	177	1.86	
Missing data	4	0.15	11	0.12	
Total	2682		9527		12209
**Times of pregnancy**					<0.001
0	601	22.41	2154	22.61	
1	666	24.83	1996	20.95	
2	607	22.63	1872	19.65	
3	400	14.91	1492	15.66	
4	233	8.69	955	10.02	
≥5	170	6.34	1038	10.90	
Missing data	5	0.19	20	0.21	
Total	2682		9527		12209
**Times of birth**					<0.001
0	604	22.52	1963	20.60	
1	1415	52.76	3970	41.67	
2	534	19.91	2106	22.11	
3	94	3.50	852	8.94	
4	24	0.89	377	3.96	
≥5	6	0.22	243	2.55	
Missing data	5	0.19	16	0.17	
Total	2682		9527		12209
**Breastfeeding history**					<0.001
Yes	982	36.61	3650	38.31	
No	137	5.11	265	2.78	
Missing data	1563	58.28	5612	58.91	
Total	2682		9527		12209

### Comparison of clinicopathological features and biomarker expression levels between the two age groups

Table 2 shows the significant differences in tumor size, axillary lymph node status, and distant metastasis between young (≤40 years) and older (>40 years) breast cancer patients. In the young (≤40 years) age group, 28.52% (3.91% + 24.61%) of patients had a tumor diameter ≤2 cm. This was significantly lower (p = 0.001) than the number observed in the older (>40 years) group, i.e., 30.46% (4.20% + 26.26%) of cases. This suggests that the tumor diameter was greater in young patients at the time of diagnosis. Similarly, the probability of axillary lymph node metastases was higher in the young patients compared to the older patients (51.83%. vs. 47.92%, p = 0.006). The probability of distant metastases was also higher in the young patient group compared to the older group (1.23%. vs. 0.70%, p = 0.008). But the difference in distribution of TNM stage between two age groups didn’t reach statistical significance (p = 0.226). Similarly, we observed a tendency in the tumor grading in young patients (i.e., the grade was higher) compared to older patients, but this was not statistically significant (p = 0.052). Additionally, we found no significant differences between the two age groups in the other clinicopathological features, such as tumor site, pathological diagnosis, and so on (data not listed).

**Table 2:** Comparison of clinicopathological features and biomarker expression levels (immunohistochemical detection) between young (≤40 years) and older (>40 years) female breast cancer patients.

Characteristic	≤40 years		>40 years		*P*-value
	N	%	N	%	
**Tumor size (cm)**					0.001
≤1	105	3.91	400	4.20	
>1, ≤2	660	24.61	2502	26.26	
>2, ≤5	957	35.68	3614	37.93	
>5	114	4.25	273	2.87	
Missing data	846	31.54	2738	28.74	
Total	2682		9527		12209
**Axillary lymph node metastasis**					0.006
Yes	1390	51.83	4565	47.92	
No	1052	39.22	3922	41.17	
Missing data	240	8.95	1040	10.92	
Total	2682		9527		12209
**Distant metastasis**					0.008
Negative	2594	96.72	9226	96.84	
Positive	33	1.23	67	0.70	
Missing data	55	2.05	234	2.46	
Total	2682		9527		12209
**TNM stage**					0.226
0	12	0.45	34	0.36	
I	344	12.83	1460	15.32	
II	856	31.92	3194	33.53	
III	339	12.64	1304	13.69	
IV	20	0.75	56	0.59	
Missing data	1111	41.42	3479	36.52	
Total	2682		9527		12209
**Tumor grade**					0.052
I	80	2.98	399	4.19	
II	721	26.88	3019	31.69	
III	323	12.04	1186	12.45	
Missing data	1558	58.09	4923	51.67	
Total	2682		9527		12209
**ER status**					0.827
Positive	1503	56.04	5338	56.03	
Negative	892	33.26	3135	32.91	
Missing data	287	10.70	1054	11.06	
Total	2682		9527		12209
**PR status**					<0.001
Positive	1445	53.88	4677	49.09	
Negative	939	35.01	3740	39.26	
Missing data	298	11.11	1110	11.65	
Total	2682		9527		12209
**ER+/PR+**					0.010
Yes	1271	47.39	4242	44.53	
No	1098	40.94	4133	43.38	
Missing data	313	11.67	1152	12.09	
Total	2682		9527		12209
**ER+/PR-**					<0.001
Yes	218	8.13	1035	10.86	
No	2151	80.20	7340	77.04	
Missing data	313	11.67	1152	12.09	
Total	2682		9527		12209
**ER-/PR+**					<0.001
Yes	167	6.23	417	4.38	
No	2202	82.10	7958	83.53	
Missing data	313	11.67	1152	12.09	
Total	2682		9527		12209
**HER2 status**					0.002
Positive	294	10.96	1194	12.53	
Negative	1331	49.63	4403	46.22	
++*	392	14.62	1541	16.18	
Missing data	665	24.79	2389	25.08	
Total	2682		9527		12209

*: Immunohistochemical testing equivocal status.

As to biomarker expression levels, there were significant differences in progesterone receptor (PR) and HER2 expression levels in breast cancer tissues of different age groups (Table 2). The PR positive expression rate was higher in young (≤40 years) patients compared with older (>40 year) patients (53.88%. vs. 49.09%, p < 0.001; Table 2). In addition, the HER2 negative expression rate was higher in the young age group compared with the older age group (49.63%. vs. 46.22%, p = 0.002; Table 2).

As double positive expression of the estrogen receptor (ER) and PR (i.e., ER+/PR+) can indicate a better endocrine therapy response, we performed a combined analysis of ER and PR expression. The ER+/PR+ double positive expression rate was higher in young patients than older patients (47.39% vs. 44.53%, p = 0.010; Table 2). Young patients also had a higher PR (ER-/PR+) single positive expression rate (6.23%. vs. 4.38%, p < 0.001), and lower ER (ER+/PR-) single positive expression rate compared with older patients (8.13%. vs. 10.86%, p < 0.001; Table 2). However, no significant differences in total ER expression levels were found between the two groups.

### Comparison of treatment modes between the two age groups

Women of different ages may have a different understanding of the disease, which could affect their treatment choices. We found that the the proportion of patients receiving surgical treatment was significantly higher in the young (≤40 years) age group compared to the older (>40 years) age group (97.58% vs. 96.60%, p < 0.05; Table 3). However, in terms of the choice of operation method, the proportion of patients receiving radical surgery (mastectomy plus axillary lymph node dissection) was lower in young patients compared to older patients (83.09% vs. 89.81%, p < 0.001; Table 3). On the other hand, a higher proportion of young women received advanced surgery (breast-conserving surgery, sentinel lymph node biopsy, sentinel lymph node biopsy plus simple mastectomy) compared to women aged >40 years (4.36%. vs. 1.77%, p<0.001; Table 3).

**Table 3:** Comparison of treatment modes between young (≤40 years) and older (>40 years) female breast cancer patients.

Characteristic	≤40 years		>40 years		P-value
	N	percent	N	percent	
**Operation**					0.011
Yes	2617	97.58	9203	96.60	
No	65	2.42	324	3.40	
Total	2682		9527		12209
**Types of operation**					<0.001
Radical surgery	2251	83.09	8505	89.81	
Advanced surgical options *	118	4.36	168	1.77	
Others**	340	12.55	797	8.42	
Total***	2709		9470		12179
**Neoadjuvant chemotherapy**					<0.001
Yes	1024	38.18	3067	32.19	
No	1614	60.18	6222	65.31	
Missing data	44	1.64	238	2.50	
Total	2682		9527		12209
**Adjuvant chemotherapy**					<0.001
Yes	2408	89.78	7957	83.52	
No	221	8.24	1259	13.22	
Missing data	53	1.98	311	3.26	
Total	2682		9527		12209
**Types of adjuvant chemotherapy**					<0.001
Taxol regimen	949	39.41	2797	35.15	
Non-Taxol regimen	1459	60.59	5160	64.85	
Total	2408		7957		10365
**Radiotherapy**					<0.001
Yes	735	27.40	1837	19.28	
No	1923	71.70	7533	79.07	
Missing data	24	0.89	157	1.65	
Total	2682		9527		12209
**Endocrine therapy**					<0.001
Yes	920	34.30	2746	28.82	
No	1738	64.80	6624	69.53	
Missing data	24	0.89	157	1.65	
Total	2682		9527		12209

*: advanced surgical procedures include: breast-conserving surgery, sentinel lymph node biopsy, sentinel lymph node biopsy plus simple mastectomy.

**: other surgical procedures include: surgical procedures except breast-conserving surgery, sentinel lymph node biopsy, sentinel lymph node biopsy plus simple mastectomy.

***: the reason the sum of surgical procedures is more than the sum of patients is due to the fact that some patients first received a sentinel lymph node biopsy, and subsequently underwent radical surgery because of the metastasis in the sentinel lymph node, and we calculated two procedures at the same time.

The implementation rates of preoperative neoadjuvant chemotherapy and postoperative adjuvant chemotherapy were significantly higher (p < 0.001) in young patients than older patients (i.e., 38.18% vs. 32.19% and 89.78% vs. 83.52%, respectively; Table 3). In terms of the choice of chemotherapy regimens, more young (≤40 years) patients selected that taxol (T) regimen in postoperative adjuvant chemotherapy compared to those in the older age group (39.41%. vs. 35.15%, p < 0.001; Table 3). However, there was no significant difference in the choices of preoperative neoadjuvant chemotherapy (data not listed). In terms of treatment with herceptin (trastuzumab), a targeted therapy for HER2-positive breast cancer, the utilization rate in both age groups was relatively low, and without significant difference. The implementation rate of radiotherapy and endocrine therapy was significantly higher (p < 0.001) in young patients compared with older patients (i.e., 27.40%. vs. 19.28% and 34.30% vs. 28.82%, respectively; Table 3).

### Multivariate logistic regression analysis of “young (≤40 years)” age breast cancer-related risk factors among all breast cancer patients

Multivariate logistic regression analysis indicated all the estrogen-related risk factors were related to “young” (≤40 years) age breast cancer: age at menarche, marital status, times of pregnancy, times of birth, breastfeeding history (Table 4). Compared with referent (age at menarche ≤10; married status; absence of a history of pregnancy and birth; breastfeeding history), (1) increase of age at menarche, divorced/widow, times of pregnancy ≥1, times of birth ≥3 were associated with decreased “young” (≤40 years) age disease possibility (OR<1, p < 0.05); (2)and never married/single, times of birth = 1, absence of a history of breastfeeding were associated with elevated “young” (≤40 years) age disease possibility (OR>1, p < 0.05) among all breast cancer patients.

**Table 4:** Multivariate logistic regression analysis of “young (≤40 years)” age breast cancer-related risk factors among all breast cancer patients.

Factors	*b*	*S*_*b*_	*X*^*2*^	P-value	*OR(95%CI)*
**Age at menarche (years)**					
≤10*					
11–12	-0.99	0.07	229.68	<0.001	0.37(0.33~0.42)
13–14	-1.11	0.04	730.84	<0.001	0.33(0.30~0.36)
15–16	-1.54	0.05	877.83	<0.001	0.21(0.19~0.24)
17–18	-1.99	0.10	411.53	<0.001	0.14(0.11~0.17)
≥19	-2.17	0.22	96.22	<0.001	0.11(0.07~0.18)
**Marital status**					
Married*					
Never married/single	2.00	0.24	68.90	<0.001	7.36(4.59~11.79)
Divorced/widow	-0.69	0.23	9.38	0.000	0.50(0.32~0.78)
**Times of pregnancy**					
0*					
1	-0.14	0.06	4.97	0.030	0.87(0.77~0.98)
**Times of birth**					
0*					
1	0.26	0.05	23.52	<0.001	1.30(1.17~1.45)
3	-0.84	0.11	53.81	<0.001	0.43(0.34~0.54)
4	-1.40	0.21	42.70	<0.001	0.25(0.16~0.38)
≥5	-2.29	0.42	30.52	<0.001	0.10(0.05~0.23)
**Breastfeeding history**					
Yes*					
No	0.39	0.11	12.05	0.000	1.48(1.19~1.84)

* Referent

Non-significant (p>0.05) data were not listed.

## Discussion

In this study, the median age at diagnosis was 48 years for all breast cancer patients. The young patients (≤40 years) accounted for 21.97% of the total breast cancer cases, which is similar to other Asian countries, such as South Korea [14, 15], Iran [16] and Saudi Arabia [17], but is significantly higher than that of western population (i.e., around 5%) [7, 8]. This suggests the possibility that certain differences in the pathogenesis of breast cancer may exist between young Chinese women and women in the western population. These differences may be related to race, social background factors, dietary habits, economic development levels, among others [16, 18–20].

Further research results in our study supported this possibility. First, we found a slightly lower ER-positive expression rate in young patients compared with older patients, but the PR-positive expression rate was significantly increased in young breast cancer patients, which differed from the results of western population. To date, most studies from western developed countries have shown that the positive expression rate of ER and PR was lower in young patients [3, 19, 21–24]. Second, As PR-positive expression reflects complete estrogen signaling pathways [25, 26], and patients with ER+/PR+ double positive expression seem to have better prognosis in the clinic [27, 28]. Therefore, we analyzed both ER and PR expression and found that the ER+/PR+ double positive rate of young patients was higher than in older patients, which was also a rare phenomenon. Third, in our study we found that the HER2 negative expression rate in young patients was higher than that of older patients. This contradicts most previous research that has found that breast cancer cells of young patients are more likely to show HER2 positive expression [3, 24, 29, 30]. The above results indicated the possibility that the pathogenesis mechanism of young female breast cancer in West China may differ from other ethnic populations in other regions, especially, western developed regions.

Of course, we could not rule out other technical or statistics factors that lead to the different results. Such as, differences in ER, PR and HER2 detection and determination methods; and in our study we were missing up to 40% of the data for HER2 expression (i.e., missing 25% original data and 15% were HER2 immunohistochemical positive “++” but without fluorescence in situ hybridization examination), which may cause bias in our results. However, the results of another study, performed in Taiwan population, supported our hypothesis. The author found similar expression patterns of ER, PR and ER+/PR+ double positive in young female breast cancer patients in Taiwan. The author suggested that the unique association of young female breast cancer in Taiwan with favorable pathological features and outcomes provides strong evidence that this population involves an emerging, distinct disease entity and should not be regraded as just a mirror image of its Western counterpart.[20]

We further analyzed the breast cancer risk factors that might be related to ER- and PR-positive expression, which may lead to present unique expression pattern of ER and PR in our study. Both the univariate and multivariate analyses showed that, among all breast cancer patients, earlier age of menarche, lower marriage rates, fewer pregnancies and births, and lower breastfeeding rates were associated with increased possibility of “young” age disease. These risk factors are closely linked to estrogen exposure, and estrogen-induced tumorigenesis of breast cancer may be induced through the ER pathway [20, 31, 32]. The emergence of these risk factors for increased estrogen exposure may be related to life habits, dietary habits, employment pressure and other social factors, as well as the specific family planning policies effective in China during different time periods. All of these factors from West China population may differ from western developed regions and contribute to different pathogenesis mechanism.

As to other clinicopathological differences between young and older female patients, we found similar results with other studies[1–3, 16, 18, 33–35]. For example, We found that the tumor diameter of young patients, at diagnosis, was larger, with a higher axillary lymph node metastasis rate, and a higher distant metastasis rate. The later tumor stage at diagnosis might be related to delayed diagnosis in young patients, which can be explained by work pressures or a low index of “suspicion” by the patient and the primary physician[33, 34]. But it was doubtful that the difference in distribution of TNM stage between two age groups didn’t reach statistical significance. A possible explanation was that we were missing up to about 35% of the data for TNM stage in both age groups.

In terms of treatment options, we compared the two age groups in terms of four aspects: surgery, chemotherapy, radiotherapy and endocrine therapy. First, in the choice of surgical approach, the proportion receiving advanced operation methods such as breast-conserving surgery and sentinel lymph node biopsy, was higher in young patients, which was consistent with our previous research [36]. This reflects previous findings that showed young patients have a greater desire to keep their original breasts, and are more accepting of advanced operation methods, compared to older patients [37].

Second, we found a higher proportion of young patients received neoadjuvant chemotherapy and adjuvant chemotherapy, which is consistent with the results of other scholars [19, 29, 30, 38]. The main reason for this difference is that adjuvant chemotherapy is more beneficial in young patients, as it significantly reduces local recurrence risk and mortality risk [38–40]. With respect to the choice of chemotherapy regimens, more young patients preferred a multi-drug combination regimen, contained “T”, for postoperative adjuvant chemotherapy. Indeed, multi-drug combination chemotherapy is suggested to be more effective than single-agent chemotherapy for young breast cancer patients, and doxorubicin-taxol combination is reported to be the most effective [41].

Third, in terms of radiotherapy, the implementation ratio of young patients was higher, which is consistent with the study by Morrison et al. [19, 38]. The increased use of radiotherapy may be due to the fact that young patients were at later local stages at the time of diagnosis, and thus, radiation therapy can effectively reduce local recurrence rate. In addition, as radiotherapy is necessary after breast-conserving surgery and the proportion of young patients receiving breast-conserving surgery was higher, this may be another factor for its increased use in young breast cancer patients.

Finally, while some previous studies have shown that the proportion of young patients receiving postoperative endocrine therapy is lower [24, 29, 42], we found the opposite. That is, the proportion of young patients receiving endocrine therapy was significantly higher than older patients in this study. In most previous studies, there were more ER/PR negative expression cases of breast cancer in the young patients, which do not require endocrine therapy. However, the young female breast cancer patients in West China showed higher PR positive and ER+/PR+ double positive expression rates. As related studies have shown that ER/PR-positive breast cancer patients under the age of 40 years should receive endocrine therapy [43, 44], this factor could reasonably explain the results observed in this study.

There are some limitations of this research. First, about 20% of breast cancer cases in young patients are related to family history, but we failed to evaluate the family history conditions for all patients in this study. Second, we only investigated the morbidity situation of female breast cancer in nine provinces of West China, which does not fully represent the morbidity situation of China as a whole. Third, this study failed to give follow-up to all patients, and therefore, the effects of relevant factors on disease free survival and overall survival are unclear. This weakens our ability to make any strong conclusions about morbidity between the age groups. Relevant follow-up is currently in progress, and therefore, the results described in this study will be validated in further research.

## Conclusions

We found significant differences in the clinicopathological features, risk factors and treatment modes between young (≤40 years) and older (>40 years) female breast cancer patients in West China. As some of these results differ from those found in the western female population, it is likely that the mechanism of tumorigenesis of young female breast cancer patients in West China may differ from that in western developed countries. Further investigation into the regional differences in breast cancer tumorigenesis is warranted.

## Supporting Information

S1 Dataset 10% of original data. (XLS)

## Abbreviations

ER: estrogen receptor
PR: progesterone receptor
HER2: human epidermal growth factor receptor 2
T: taxol
